# The Association Between E-cigarette Use and Sleep Duration Among Adolescents and Young Adults: A Structured Narrative Review

**DOI:** 10.7759/cureus.112672

**Published:** 2026-07-14

**Authors:** Nishita Mishra, Rajal Mehta

**Affiliations:** 1 Community Medicine, BASIS Scottsdale, Scottsdale, USA; 2 Internal Medicine, Mayo Clinic, Scottsdale, USA

**Keywords:** adolescents, e-cigarettes, sleep duration, vaping, young adults

## Abstract

E-cigarette use has become increasingly widespread among youth, raising concerns that nicotine exposure may contribute to sleep disruption. This structured narrative review synthesizes current evidence on the association between e-cigarette use and sleep duration among adolescents and young adults. Studies were evaluated using predefined inclusion criteria, and eligible studies examined e-cigarette use alongside sleep duration or related sleep disturbances in youth populations. Extracted data included study design, population characteristics, exposure definitions, sleep measures, and statistical associations, while study quality was assessed based on sample representativeness, outcome validity, and adjustment for confounding variables. Six empirical studies and one narrative review met the inclusion criteria. Across large national datasets, exclusive e-cigarette use was consistently associated with 30-80% higher odds of insufficient sleep among adolescents and young adults, while dual users demonstrated the strongest associations and were up to three times more likely to report inadequate sleep. Adolescents using both combustible and electronic cigarettes also showed increased sleep latency. Emerging evidence suggests a potential bidirectional relationship in which sleep problems may increase susceptibility to vaping initiation. Overall, current evidence indicates that e-cigarette use is repeatedly linked to inadequate sleep duration and other sleep difficulties in youth. Nicotine’s neurobiological effects on arousal and withdrawal likely contribute to these outcomes, while sleep disturbances themselves may also increase vulnerability to nicotine use. Public health strategies aimed at reducing youth vaping may therefore improve sleep health, and interventions targeting sleep may help reduce nicotine use. Longitudinal and mechanistic studies are needed to clarify causal pathways and underlying processes.

## Introduction and background

Introduction

Sleep is essential for healthy physical, cognitive, and emotional development during adolescence and young adulthood. Emerging evidence suggests that health behaviors, including nicotine use through electronic cigarettes, may adversely affect sleep. This review summarizes the evidence examining the relationship between e-cigarette use and sleep outcomes among adolescents and young adults. Unlike previous reviews that have broadly examined the health effects of e-cigarette use or summarized sleep-related outcomes, this structured narrative review specifically synthesizes the current evidence on the relationship between e-cigarette use and sleep outcomes among adolescents and young adults, critically evaluates methodological strengths and limitations of the available literature, and identifies priorities for future research and clinical practice.

Background

Insufficient sleep has become a universal concern for both young adults and adolescents, since it impacts various aspects of physical, emotional, and mental health. According to national estimates, the majority of youth fail to meet age-based sleep recommendations, and many habitually sleep fewer than eight hours on school nights, a level associated with heightened risks for academic difficulties, emotional dysregulation, and behavioral health concerns [1,2]. Recent studies have consistently identified associations between e-cigarette use and decreased sleep duration and other adverse sleep outcomes in both adolescents and young adults [1-6]. For instance, analyses of the Youth Risk Behavior Survey data have shown that current users of electronic vaping products have significantly higher odds of insufficient sleep compared with never-users [1,2]. As the frequency of sleep disturbances continues to grow, emerging behaviors, such as the rapid uptake of electronic cigarette use, may be contributing factors that demand further attention [5]. Because of both the importance of adequate sleep during adolescence and young adulthood and the rising prevalence of nicotine vaping, a thorough analysis of the available evidence is essential. This review summarizes current evidence on the relationship between e-cigarette use and sleep patterns in youth, highlights the strengths and limitations of existing studies, and outlines considerations for future research and clinical practice.

## Review

Literature search and study selection

A structured narrative review methodology was used to identify and synthesize evidence examining the relationship between e-cigarette use and sleep outcomes among adolescents and young adults. Relevant literature was identified through searches of PubMed/MEDLINE and Google Scholar for studies published through June 2025 using combinations of keywords, including "e-cigarette," "electronic cigarette," "vaping," "electronic nicotine delivery systems (ENDS)," "sleep," "sleep duration," "sleep quality," "sleep disturbance," "insomnia," "adolescents," and "young adults." Reference lists of eligible articles were also screened to identify additional relevant studies. Eligible studies included adolescents or young adults and evaluated e-cigarette use, electronic vaping products, or electronic nicotine delivery systems (ENDS) in relation to sleep duration, sleep quality, sleep latency, insomnia symptoms, or sleep-related complaints. Cross-sectional surveys, cohort studies, and secondary analyses of national datasets were included in the primary evidence synthesis. Systematic reviews, meta-analyses, and narrative reviews were used to identify additional eligible primary studies and to provide contextual background, but were not included as independent evidence in the primary synthesis to avoid duplication of primary studies. Studies focused exclusively on combustible tobacco without a separate assessment of e-cigarette exposure or those lacking relevant sleep outcomes were excluded. Information extracted from each study included study design, sample characteristics, exposure definitions, sleep outcomes, statistical associations, major findings, and methodological limitations. Quality assessment focused on sample representativeness, validity of sleep and exposure measures, adjustment for confounding variables, and ability to support causal inference. Most studies used large nationally representative datasets but relied on self-reported sleep and vaping measures.

Review of evidence

The characteristics and principal findings of the included studies are summarized in Table 1. Across studies, e-cigarette use was consistently associated with adverse sleep outcomes, including short sleep duration, poor sleep quality, delayed sleep onset, insomnia symptoms, and daytime dysfunction. Nationally representative analyses from the YRBS (Youth Risk Behavior Survey), BRFSS (Behavioral Risk Factor Surveillance System), NHIS (National Health Interview Survey), and PATH (Population Assessment of Tobacco and Health) datasets demonstrated higher odds of inadequate sleep among current e-cigarette users compared with non-users, with the strongest associations observed among dual users of combustible cigarettes and e-cigarettes.

**Table 1:** Summary of studies evaluating the association between e-cigarette use and sleep outcomes among adolescents and young adults. Abbreviations: YRBS, Youth Risk Behavior Survey; BRFSS, Behavioral Risk Factor Surveillance System; NHIS, National Health Interview Survey; VAYPS, Virginia Youth Prevention Survey; ENDS, electronic nicotine delivery systems; OR, odds ratio.

Study	Population	Data source/Design	Sleep outcome	Main findings
Baiden et al., 2023 [1]	Adolescents	YRBS 2017-2019	<8 hours	Current and former vaping increased the odds of insufficient sleep.
Merianos et al., 2021 [2]	Adolescents	YRBS 2017	<7 or <8 hours	Exclusive and dual users had the highest odds of short sleep.
Li et al., 2025 [3]	Young adults	BRFSS 2022	Short sleep duration	Exclusive e-cigarette users had increased odds of short sleep duration.
Merianos et al., 2023 [4]	Young adults	National survey analysis	Inadequate sleep duration	Exclusive e-cigarette users, cigarette smokers, and dual users had higher odds of inadequate sleep.
Sulthana et al., 2025 [5]	Mixed adolescent/adult samples	Systematic review and meta-analysis	Short sleep and sleep disturbance	E-cigarette users had higher odds of short sleep; pooled OR = 1.38.
Poudel et al., 2025 [6]	Young adults (18-35)	NHIS 2020	Short sleep, sleep quality, sleep medication	Dual users had the greatest odds of poor sleep and sleep disturbance.
Malhotra et al., 2021 [7]	Adolescents	Online survey	Sleep latency	Dual users had prolonged sleep latency.
Sutherland et al., 2022 [8]	Adolescents	Longitudinal neuroimaging sample	Sleep problems and depressive symptoms	Amygdala-insula connectivity was associated with sleep problems, depressive symptoms, and later e-cigarette use.
Welty et al., 2023 [9]	Adolescents	YRBS 2019	Sleep <7 hours and suicidality	Vaping and short sleep were associated with higher odds of suicidal thoughts, plans, or attempts.
Birur et al., 2025 [10]	Adolescents	Narrative review	Sleep duration and latency	Evidence supports a bidirectional association between sleep problems and nicotine vaping.
Langley et al., 2025 [11]	Commentary/Policy review	Narrative commentary	Public health implications	Discusses rising adolescent e-cigarette use, nicotine concentrations, marketing, and long-term health concerns.
Holtz et al., 2022 [12]	Adolescents	Cross-sectional VAYPS	Sleep deprivation and ENDS susceptibility	Less than 6 hours of sleep was associated with greater odds of susceptibility to vaping initiation.

Among adolescents, several studies reported a consistent relationship between vaping and insufficient sleep. Baiden et al. found that current use of electronic vaping products was associated with greater odds of insufficient sleep [1]. Merianos et al. reported that exclusive e-cigarette users were more likely to sleep fewer than the recommended number of hours, with stronger associations among dual users [2].

Similar findings have been reported among young adults. Li et al. found that exclusive e-cigarette users had increased odds of short sleep duration [3]. Merianos et al. demonstrated that exclusive e-cigarette users, combustible cigarette users, and dual users all experienced higher odds of inadequate sleep relative to non-users [4].

Recent evidence synthesis supports these findings. Sulthana et al. reported a pooled odds ratio of 1.38 for shorter sleep duration among e-cigarette users and additionally identified increased insomnia symptoms, trouble sleeping, daytime dysfunction, and poor sleep quality [5].

Poudel et al. showed that dual users had the greatest odds of poor sleep and more frequent trouble falling asleep, trouble staying asleep, non-restorative sleep, and sleep medication use [6]. Malhotra et al. identified prolonged sleep latency among adolescents using both cigarettes and e-cigarettes, suggesting that nicotine exposure may impair sleep initiation in addition to reducing total sleep time [7].

Across large national datasets, exclusive e-cigarette use was consistently associated with higher odds of insufficient sleep among adolescents and young adults, with adjusted odds ratios (AORs) ranging from 1.33 (95% CI, 1.16-1.52) to 1.82 (95% CI, 1.30-2.54), while dual users demonstrated the strongest associations (AOR, 2.03; 95% CI, 1.44-2.86) [1-7].

Emerging evidence also suggests important neurobiological and psychosocial interactions between sleep disturbance, mental health, and vaping behavior. Sutherland et al. found that greater amygdala-ventral insula functional connectivity predicted increased sleep problems, which were associated with greater depressive symptoms and subsequent e-cigarette use [8]. Welty et al. reported that both vaping and short sleep were independently associated with increased odds of adolescent suicidality, and that adolescents who both vaped and obtained fewer than seven hours of sleep on school nights had markedly increased odds of suicidal ideation and suicide attempts [9].

Despite the consistency of these findings, interpretation should be cautious. Most available studies are cross-sectional and therefore cannot establish causality. It remains unclear whether vaping contributes to sleep disruption, whether sleep problems increase vulnerability to vaping initiation, or whether both are driven by shared factors such as anxiety, depression, stress, screen use, substance use, and family or peer influences. Birur et al. highlighted this likely bidirectional relationship, proposing that insufficient sleep may predispose adolescents to nicotine vaping while vaping itself worsens sleep quality and duration, creating a self-reinforcing cycle [10].

The broader public health implications are also noteworthy. Contemporary commentaries and policy reviews have raised concerns regarding the rapid rise of adolescent e-cigarette use, increasing nicotine concentrations in newer devices, youth-targeted marketing, and uncertainty regarding long-term health effects [11]. Holtz et al. found that adolescents sleeping fewer than six hours per night had higher odds of susceptibility to initiating ENDS use, suggesting a potentially bidirectional relationship [12]. Emerging evidence additionally suggests that vaping may contribute to respiratory, neurocognitive, and mental health disturbances that can independently affect sleep and overall well-being.

Emerging evidence additionally suggests that vaping may contribute to respiratory, neurocognitive, and mental health disturbances that can independently affect sleep and overall well-being.

Discussion

Current evidence consistently demonstrates an association between e-cigarette use and adverse sleep outcomes among adolescents and young adults. Across multiple large observational studies and nationally representative datasets, vaping has been associated with shorter sleep duration, poor sleep quality, insomnia symptoms, delayed sleep onset, and daytime dysfunction. These associations have been observed among both adolescent and young adult populations, with dual users of combustible cigarettes and e-cigarettes consistently demonstrating the greatest risk of sleep-related impairment [1-7]. Recent meta-analytic evidence further supports these findings, reporting increased odds of short sleep duration, insomnia symptoms, trouble sleeping, daytime dysfunction, and poor sleep quality among e-cigarette users [5]. Recent reviews have further emphasized the growing public health burden of adolescent vaping and highlighted the need for additional longitudinal research to better understand its effects on neurobehavioral outcomes, including sleep and mental health [13,14].

Several biological mechanisms may explain this association. Nicotine acts as a central nervous system stimulant by activating nicotinic acetylcholine receptors, leading to increased release of neurotransmitters such as dopamine, norepinephrine, acetylcholine, and glutamate that promote wakefulness and physiologic arousal. These effects may delay sleep onset, reduce total sleep duration, disrupt sleep continuity, suppress slow-wave and rapid eye movement (REM) sleep, and contribute to withdrawal-related awakenings during sleep. Adolescents may be particularly vulnerable because neural systems involved in sleep regulation, reward processing, and executive functioning remain under development. Emerging neuroimaging evidence suggests that altered amygdala-insula connectivity may represent a shared pathway linking sleep disturbance, depressive symptoms, and e-cigarette use [8].

An important finding across the literature is the likelihood of bidirectional relationships between sleep and vaping behaviors. Sleep deprivation may increase susceptibility to vaping initiation through impaired decision-making, emotional dysregulation, and heightened reward sensitivity, while nicotine exposure may worsen sleep duration and quality through direct neurophysiologic effects. Together, these processes may create a self-reinforcing cycle that contributes to the persistence of both sleep disturbances and nicotine use among adolescents and young adults [10].

The clinical and psychosocial implications are substantial. Beyond sleep-related symptoms, vaping and inadequate sleep have each been associated with adverse mental health outcomes, including depressive symptoms, suicidal ideation, and suicide attempts. Evidence suggests that adolescents who both vape and experience insufficient sleep may represent a particularly vulnerable population at elevated risk for psychological distress and self-harm behaviors [8,9]. These findings highlight the importance of assessing sleep health when counseling youth who use nicotine products and suggest that sleep-focused interventions may complement nicotine cessation efforts.

The existing literature possesses several strengths, including large sample sizes, nationally representative datasets, and consistent findings across diverse populations and study designs [1-7]. However, important limitations remain. Most studies are cross-sectional and therefore cannot establish temporal relationships or causality. In addition, the majority rely on self-reported measures of both vaping behavior and sleep outcomes, which may be subject to recall and reporting bias. Many studies also provide limited characterization of vaping frequency, device type, nicotine concentration, and duration of use. Consequently, it remains unclear whether vaping directly causes sleep disruption, whether poor sleep predisposes individuals to nicotine use, or whether both are influenced by shared factors such as anxiety, depression, stress, screen use, substance use, and family or peer environments [10]. Sleep outcomes were also assessed using heterogeneous measures across studies, including differing definitions of insufficient sleep, sleep duration thresholds, sleep quality, sleep latency, insomnia symptoms, and daytime dysfunction, which may limit direct comparisons of findings.

Future research should prioritize longitudinal studies capable of clarifying causal pathways, incorporate objective sleep assessments and biochemical verification of nicotine exposure, and more comprehensively characterize vaping behaviors. Improved understanding of these relationships may help inform interventions aimed at reducing both nicotine use and sleep disturbances among adolescents and young adults.

## Conclusions

Overall, the body of evidence supports a consistent association between e-cigarette use and poorer sleep outcomes among adolescents and young adults, including insufficient sleep duration, prolonged sleep latency, daytime dysfunction, and other sleep-related complaints. Across studies, dual users of combustible cigarettes and e-cigarettes appear to experience the greatest degree of sleep disruption, suggesting a possible exposure-response relationship related to cumulative nicotine burden and dependence severity. However, the available evidence is derived predominantly from observational, largely cross-sectional studies, and therefore, causal relationships cannot be established. Although the findings collectively suggest a likely bidirectional relationship in which nicotine vaping may worsen sleep while chronic sleep deprivation may also increase vulnerability to vaping initiation and continued use, this hypothesis requires confirmation in longitudinal and mechanistic studies. Given the critical role of sleep in adolescent neurodevelopment, emotional regulation, academic functioning, and mental health, these findings have important clinical and public health implications. Integrating sleep education and sleep health screening into vaping prevention and cessation programs may offer a valuable opportunity for early intervention. Clinicians caring for adolescents and young adults should routinely assess both nicotine use and sleep behaviors concurrently, as addressing one domain may positively influence the other. Future research should prioritize well-designed longitudinal and mechanistic studies using objective sleep measures and detailed characterization of vaping behaviors, including nicotine concentration, device type, frequency, and timing of use, to clarify temporal relationships, establish causality, and better understand the long-term effects on sleep health.
